# Possible generation of heat from nuclear fusion in Earth’s inner core

**DOI:** 10.1038/srep37740

**Published:** 2016-11-23

**Authors:** Mikio Fukuhara

**Affiliations:** 1 New Industry Creation Hatchery Centre, Tohoku University, Sendai, 980-8579, Japan; 2 Waseda University Research Organization for Nano & Life Innovation, Green Device Laboratory, Tokyo, Japan

## Abstract

The cause and source of the heat released from Earth’s interior have not yet been determined. Some research groups have proposed that the heat is supplied by radioactive decay or by a nuclear georeactor. Here we postulate that the generation of heat is the result of three-body nuclear fusion of deuterons confined in hexagonal FeDx core-centre crystals; the reaction rate is enhanced by the combined attraction effects of high-pressure (~364 GPa) and high-temperature (~5700 K) and by the physical catalysis of neutral pions: ^2^D + ^2^D + ^2^D → 2^1^H + ^4^He + 2 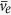 + 20.85 MeV. The possible heat generation rate can be calculated as 8.12 × 10^12^ J/m^3^, based on the assumption that Earth’s primitive heat supply has already been exhausted. The H and He atoms produced and the anti-neutrino 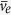 are incorporated as Fe-H based alloys in the H-rich portion of inner core, are released from Earth’s interior to the universe, and pass through Earth, respectively.

Our earth is still a young planet with substantial heat sources that are characterised by volcanic activity and earthquakes generated by the movement of tectonic plates. The theory of plate tectonics successfully explains various geological phenomena that occur on the continents and in the oceans of Earth, but the driving force behind plate motion has not been entirely resolved. Regarding origin of the heat, the current consensus is that the flow of heat from Earth’s interior to the surface comes from two main sources: radiogenic heat and primordial heat. Primordial heat, which was generated during initial formation of Earth, is the kinetic energy transferred to Earth by external impacts of comets and meteorites and the subsequent effects: gravity-driven accretion, friction caused by differentiation of Earth’s mantle structure (sinking of heavy elements like Fe, rising light elements like Si) and latent heat of crystallization released as the core solidified^1^.

Since Kuroda^2^ first proposed that natural fission reactors were operating on Earth around two billion years ago, much attention has been focused on nuclear energy as the driving force of plate motion. Herndon^3^ asserted the feasibility of planetocentric nuclear reactors and developed the concept extensively. Because there is very little U in iron meteorites, however, a nuclear reactor in Earth’s core or on other terrestrial planets seems unlikely^4^. Meijer and van Westrenen^5^ reported nuclear fission of U and Th as heat generation sources at the mantle boundary within Earth’s core, based on the distribution of an isotope of Nd in rocks^6^. Bao^7^ noted that there are many heat producing elements (U and Th) in a calcium perovskite reservoir at the base of the mantle.

In 2005 and 2007, scientists at the Kamioka Liquid-Scintillator Antineutrino Detector(KamLAND)^8^ and Borexino^9^ detected signals of antineutrinos, 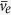 (*i.e*., “geoneutrinos”) produced inside Earth, respectively. Neutrinos, very light subatomic particles, are generated by the nuclear fission and decay of radioactive elements as well as by nuclear fusion that occurs in the sun and stars. Because fission occurs on the timescale of a fraction of a microsecond, it is necessary for heat generation to include a chain reaction of the nuclear decay of atoms in rocks and minerals with high concentrations of radioactive decaying atoms. As such, alpha and beta decay can supply heat on timescales comparable to the age of Earth. The KamLAND Collaboration reported that heat from radioactive decay of radiogenic isotopes such as ^238^U and ^232^Th contributes about half, 21 TW, of Earth’s total heat flux (44.2 ± 1.0 TW) and that Earth’s primordial heat supply has not yet been exhausted^10^.

However, there are four unanswered questions regarding the decay of such radioactive isotopes. The first question asks why a large nuclear mass emission from radioactive elements primarily concentrated in the shallow crust would not lead to the death of many living things. Although it is believed that the radiogenic heat production rate cannot cause damage to living things, we have seen an example of spontaneous ignition due to high enough concentrations of radioactive elements in crustal rocks at Oklo in Gabon, Africa^11^. In the case of spontaneous ignition, the emission products from radioactive elements would be distributed through active volcanoes and the movement of mountain ranges. Indeed, we do not suffer from natural radioactive pollution. The second question regards the amount of Pb that exists in Earth’s crust. The KamLAND Collaboration^10^ reported emission of 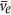 by two reactions, ^238^U → ^206^Pb + 8α + 6e^−^ + 6 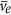 + 51.47 MeV and ^232^Th → ^208^Pb + 6α + 4e^−^ + 4 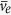 + 42.7 MeV after six- and four-times of β decay, respectively. If these reactions are responsible for the continuing heat generation in the crust, a large amount of Pb would be included in natural rocks and ores. However, the concentration of Pb in the crust is only 12.5 ppm^12^. The third question addresses the heat to thermal imbalance: the estimated slope of temperature change from the core to the crust is negatively linear. The linear slope can be explained by heat generation in Earth’s inner core only. If the estimated heat contributions from the mantle (10 TW) and the crust (7.9 TW)^10^ are correct, the temperature curve must have two peaks, one in the crust (6–40 km) and one in the mantle (410–2900 km) (Supplementary Information 1). The inhomogeneity of surface heat flow in the crust could be derived from geological disturbance in pressure-less region. As for the fourth question, if radioactive decay has also been occurring on Venus, which is Earth’s sister planet with similar size and composition, we should observe plate tectonics as a result of the carbonate magma-ocean. Plate tectonics, however, are not evident on Venus^13^. Thus, these facts put severe constraints on the possibility that radiogenic heat production in the crust and the mantle are producing 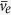.

Alternatively, we consider inductively the possibility of nuclear fusion, which does not create harmful radioactive waste but generates a large amount of heat. Because an increase in paraeomagnetic magnitude between 2.7–2.1 billion years indicates the nucleation of the inner liquid-core^14^, nuclear fusion would have started around 2.2 billion years ago^15^. Our hypothesis may explain why plate tectonics exist on Earth but not on other terrestrial planets, such as Mercury, Venus, Mars, and Earth’s moon. Furthermore, another example of nuclear fusion in Earth’s interior is that the origin of N in Earth’s atmosphere is interpreted to be the result of endothermic nuclear transmutation^16^,^17^.

## Nuclear fusion reactions without radioactivity

Labaune *et al.*^18^ demonstrated a low-energy fusion reaction of protons and ^11^B nuclei by colliding a laser-accelerated proton beam with a laser-generated B plasma. This is a cleaner and less hazardous reaction compared with hot nuclear fusion that does not produce high-energy neutrons. Hence, we introduce a low-energy nuclear fusion reaction that may be responsible for the production of thermal energy without deadly radiation.

When we first considered proton-mediated reactions, the realm of possible reactions was restricted to the following two, taking into consideration the ratio of nuclei on Earth (Supplementary Information 2):


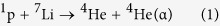



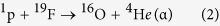


However, the small Clarke numbers of Li and F in Earth’s crust make these reactions unlikely.

Alternatively, because deuteron-mediated reactions require stable nuclides, the following reactions are promising (Supplementary Information 3).


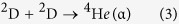



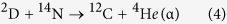



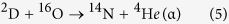


It would be difficult for reaction (4) to supply a considerable amount of N in the high-temperature and high-pressure regions required for nuclear fusion. Because the generation of atmospheric O began around 2 billion years ago as a result of photosynthetic activity by organic matter^15^, reaction (5) is also limited. Thus, reaction (3) is the most hopeful.

## Concentration of deuterium in the Fe-rich alloy core

Nuclear fusion reaction sites on Earth require the following conditions: a large quantity of deuterium (D) atoms in solid state materials, an environment with high temperature and high pressure for overcoming the high Coulomb barrier of the fusion reaction and the presence of a physical catalysis promoting the reactions. Thus Earth’s Fe-rich alloy core, with limited U and Th^3^, is a probable site. According to recent research by Tateno *et al.*^19^, the hexagonal close-packed (hcp) structure of Fe is stable up to 377 GPa and 5700 K, which corresponds to Earth’s inner core conditions. The c/a axial ratio of the hcp structure of Fe at 332 GPa and 4820 K is nearly equal to the ideal hcp structure.

Here we note the H content in the inner core. High-temperature and three-dimensional X-ray microtomographic imaging estimated 0.6 wt% (=25 at%) H in earth’s core^20^. This suggests that a large amount of H was incorporated into metals from a hydrous magma ocean at the time of core formation^21^. Baranowski^22^ showed that H atoms can migrate among Fe atoms without forming hydrides in ultra-high pressure conditions. In high temperature regions, Fukai^23^ estimated that H atoms did not remain within the potential wells of interstitial sites but experienced free motion similar to the motion of atoms in gases.

On the other hand, the contribution of D to the possible occurrence of nuclear fusion in the inner solid-core has been overlooked, although bombardment of a late veneer of comets and meteorites with H_2_O and D_2_O originating from the Kuiper belt on the primitive dry Earth has continued for 0.2 billion years after solar system isolation^24^,^25^. We note again the D content in the inner-solid core, because the D in the water-ice found in comets^26^,^27^ or meteorites^28^ is enriched by a factor of 11.6, 92, and 29, respectively, relative to the D/H ratio (0.0017) of Earth’s seawater. Thus the volume of primitive heavy water D_2_O, as estimated from the total water (1.4 billion km^3^, including Earth’s groundwater^29^) was approximately 100 million km^3^. Indeed, deuteron water exists as DHO in natural water under ambient pressure. The densities of D_2_O and DHO are 1.1056 and 1.054 g/cm^3^, respectively, and they are somewhat greater than the density of light water H_2_O (0.9982 g/cm^3^ at 293 K, 1 atm)^30^. However, if DHO sinks to the inner regions of Earth, DHO would be converted to D_2_O during the gravitational separation of D_2_O and H_2_O and then decomposed to D and O atoms. Lastly, D atoms would be incorporated into Fe-rich metals; Nomura *et al.*^20^ have estimated the Fe-H-Si system without D as core solid metals. However, research for the sinking process of the D atoms cannot be found in the literature, as far as we know.

**Figure 1:**
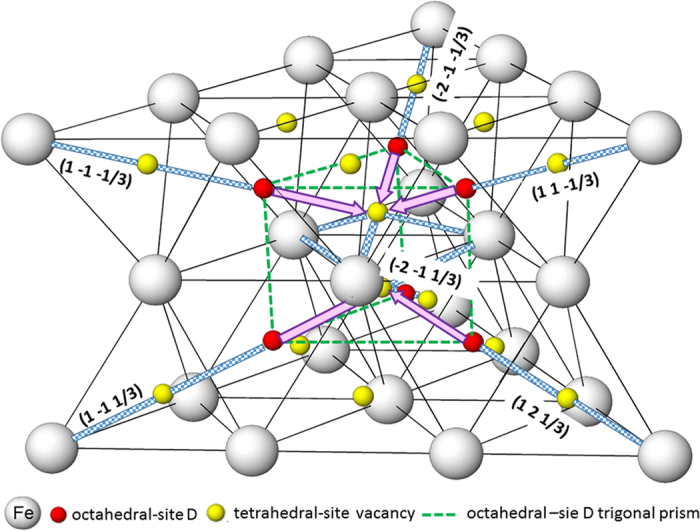
Substoichiometric FeDx crystal with all octahedral D sites (small red circles) and all tetrahedral vacancy sites (small yellow circles) in an Fe (large white circles) hexagonal close-packed (hcp) lattice at 332 GPa and 4820 K near the inner core centre. The blue thick lines represent chains (Fe−tetrahedral-site vacancy – octahedral-site D−tetrahedral-site vacancy−Fe) along [3 3 · 1] directions. The atomic sizes are not necessarily to scale.

Next we consider the driving force responsible for the close proximity of deuterons. Because maximum hydrogen solubility of the Fe-based alloy composing the centre core is 25 at% H^21^, we applied the model of the octahedral occupied deuterated iron FeDx (x < 0.25) lattice illustrated in Fig. 1, which considers the Fe-based alloy as Fe. The stable structure of metal-hydrogen alloys under high pressures and high temperatures favours interstitial sites with superabundant vacancies (vacancy-hydrogen clusters)^31^. Measurement of the defect trapping of D implanted in Fe indicates that the D atom moved from the near-octahedral interstitial site to another site^32^. The atoms along six [3 3 · 1] directions (blue thick lines) at 332 GPa and 4820 K are lined up in chains as follows: Fe―tetrahedral-site vacancy―octahedral-site D―tetrahedral-site vacancy―Fe. The interstitial deuterons in the FeDx are immersed in a sea of conduction electrons derived from Fe atoms. Charge density wave (CDW) instability occurs mostly in materials in which the atoms are lined up in chains. When hexahedral distortion of the sublattice trihedral deuterons (dotted green line) modulates the charge transfer in the chains (i.e., alternating tetrahedral D^+δ^-Fe^8-δ^ array^33^), the breathing-mode-like displacement of deuterons occurs along the [3 3 · 1] directions (Supplementary Information 4).

Thus we can infer the following dynamic nuclear reactions^34^ in which two tetrahedral-site deuteron atoms collide at the octahedral vacancy in Fe-rich alloys in Earth’s inner core:


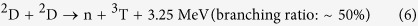



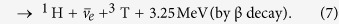


After an H atom moves to another octahedral-site, another deuteron from a neighbouring site collides with the tritium atom.


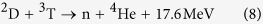



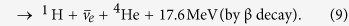


When three octahedral-site deuterons collide simultaneously at a tetrahedral vacancy site, we get the following three-body reaction,


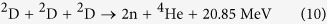



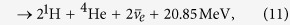


from Eqs (6) through (11). Therefore, we can measure antielectron neutrinos 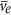 using Eq. (11). These could be the geoneutrinos that have been detected by KamLAND^10^ and Borexino^9^.

Based on both observations of a high He isotope ratio at volcanic areas^35^ and the radiation of excess heat, Jones *et al.*^36^ suggest the possibility of cold nuclear fusion in the mantle water reservoir of Earth, as well as in the core of Jupiter^4^.

## Confinement by the high-pressure effect

The outer shell electrons of D atoms in Earth’s inner core FeDx lattices behave as free electrons^37^, and the resulting screening effect provides relief from the Coulomb repulsive force between deuteron nuclei. In this study, we use a hexagonal (ε) Fe instead of an Fe-Si lattice because of the lack of crystal lattice data under high pressures around 364 GPa. The density of ε-Fe at 364 GPa and 5773 K can be estimated as 13.357 Mg/m^3^ using the density-pressure relationship^38^. Thus we can assume the tetrahedral D diameter to be 2*r*_*1*_ = 2 × 0.10395 × 0.225 ≅ 0.0468 nm (Supplementary Information 5), which is 37% less than the equilibrium diameter (2*r*_*o*_ = 0.074 nm) of a D atom in its gas or liquid phase^39^. However, this distance is still large compared to the distance (0.022 nm)^40^ required for a dynamic nuclear reaction.

## Confinement by the high-temperature effect

Because the temperature at 6378 km below the surface is reported to be about 5773 K^19^, we considered the effect of temperature on the reaction rate *k*. The rate can be expressed by the Arrhenius equation^41^:


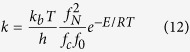


where *f*_*D*_ and *f*_*He*_ are partition functions of ^2^D and ^4^He, respectively, and *k*_*B*,_ *R* and *E* are the Boltzmann and Gas constants and the activation energy of the reaction, respectively. Because *f*_*D*_ ≒ *f*_*He*_, we can write a ratio of the rates at temperatures T_0_ and T_1_ as follows:


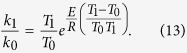


With T_0_ = 300 K and T_1_ = 5773 K, we get


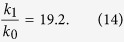


According to the first principle of the symmetry of force which is associated with a binding energy, the following potential form expresses the repulsive interaction between atoms^42^:


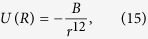


where *B* is an empirical parameter. Taking the effect of temperature on the reaction rate into consideration, we get a shrunken distance


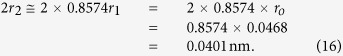


This radius is about twice the critical distance (0.022 nm).

## Physical catalysis effect for dynamic reactions of deuteron pairs

Lastly, we must consider the necessary conditions for the possible nucleonic reaction (11) followed by the formation of ^4^He. The deuteron is an *np* state whose isospin wave function is antisymmetric, where *n* and *p* are neutron and proton, respectively. The He nucleus is mediated by positively and negatively charged pions π^±^ and a single neutral pion π^o^. Therefore, the formation of the He nucleus from two deuterons (fusion) requires a direct force caused by the exchange of two neutral pions that do not compose the deuteron nucleus because the additional non-exchange part mediated by the neutral pion substantially moderates the *n, p* force in the He nucleus^39^.


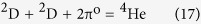


Pions are responsible for all low-energy nuclear interactions^43^ (Supplementary Information 6).

The neutral pion in Eq. (18) is provided via the fundamental process of electromagnetic interaction:


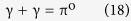


Based on isospin symmetry, the photon in Eq. (18) is produced by the emission of excited electrons e* that are generated by the collision of free electrons^44^ derived from pressure ionization (Supplementary Information 7), and cyclic expansion and contraction due to lunar gravitation,


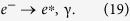


In our previous paper^39^, we reported the following formula for fusion of He without radioactivity:


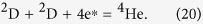


The introduction of neutral pions makes it possible to remarkably reduce the internuclear distance between deuterons, enhancing the fusion rate for He formation, as it was physical catalysis^39^.

According to the Symmetrical Meson Theory of Nuclear Force^43^ and a binding energy that tends to clump bosons together, we can note the interaction energy of two nucleons at separation *r* as follows:


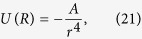


where *A* is a coupling constant. Because the addition of two neutral pions increases the attraction force by a factor of 14 in an interaction force of 14 times, we obtain the D-D distance 2*r*_*3*_.


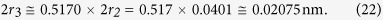


This value would lead to fusion of D-D-D nuclei before the transformation of He.

## Existence of a D-rich inner core

The fusion rate *R* for a three-body reaction without radioactive products (Eq. [11]) was calculated as 5.27 × 10^9^ fusion/s/m^3^. (Supplementary Information 8). From Eq. (11), we obtain the amount of heat generated:


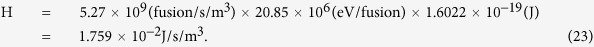


The total heat supply from the nucleation of Earth’s core to the present time (2.2 billion years^15^) is given as


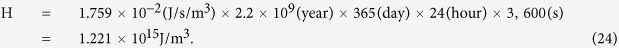


Because the current total heat flux from Earth to space is reported to be 44.2 ± 1.0 TW^45^, the total heat supply from the nucleation of Earth’s core to the present time is 3.07 × 10^30^ J (=44.2. × 10^12^ J/s × 6.94 × 10^16^ s), based on the assumption that Earth’s primitive heat supply has already been exhausted. Thus we can calculate the total D volume required for heat generation from Eq. (24),


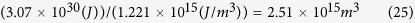


Because the volumes of ε-Fe and D in Fe-0.6 wt% D crystal (100 g) near the inner core can be estimated as 7.44 cm^3^ (≈99.4 g/13.357 g · cm^−3^) and 0.3 cm^3^ (≈0.6 g/2 g/cm^3^), respectively, the volume of Fe-D crystals that contributed to heat generation up through the present is


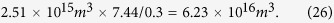


**Figure 2:**
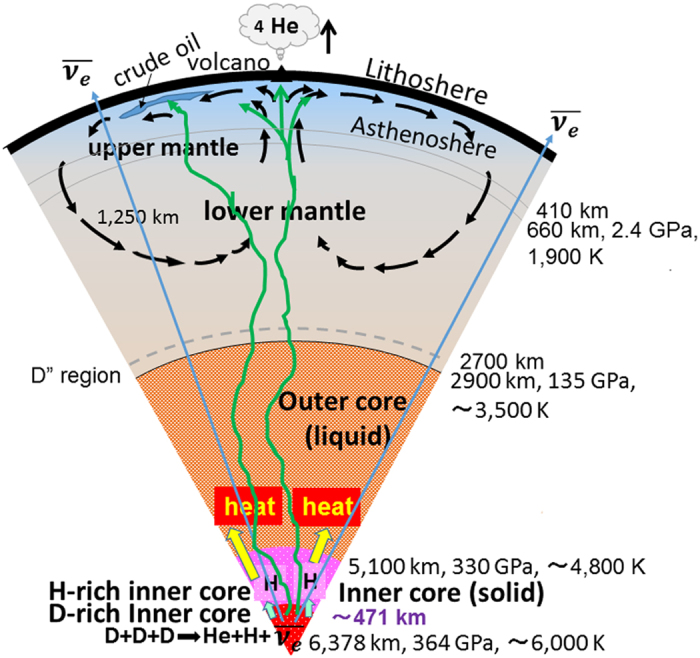
Earth’s cross-section showing the crust, upper- and lower-mantle, and outer- and inner-cores. The inner core comprises an H-rich core and a D-rich core. A substantial amount of heat is generated by nuclear dynamic fusion of deuterons squeezed in highly compressed hexagonal close-packed (hcp) Fe-rich crystal lattice near the inner core centre. The H and He atoms and the anti-neutrino 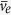 that are produced are incorporated as Fe-H based alloys in the H-rich inner core, are released from Earth’s interior to the universe, and pass through Earth, respectively.

On the other hand, because the incorporated gross weight of D from the creation of Earth’s core to present time is estimated as 6.67 × 10^21^ kg (=100 Mkm^3^ × 2 Mg/m^3^ × 4/20 × 1/0.006), the volume of Fe-D crystals up through the present is 4.99 × 10^17^ m^3^ (6.67 × 10^21^/13.36 [Mg/m^3^]), resulting in an unreacted (Fe-D) crystal volume of 4.37 × 10^17^ m^3^ (=4.99 × 10^17^–6.23 × 10^16^). Thus we can estimate 471 km (0.08 vol.% of inner core) as the radius of the D-rich inner core in Fig. 2. The heat generated is transported from the inner to the outer core and then produces mantle flow passing through the core-mantle transition zone. However, it is not clear how the heat from the inner core can reach the mantle because of its elastic anisotropy^46^ and its spatially variable heat loss^47^. Furthermore, whether the thermal circulation of the mantle has a whole-mantle or a layered-mantle configuration remains to be resolved^47^. The volatile and non-active He gases could be discharged by hydrothermal and volcanic activities and then released from Earth’s atmosphere to the universe. Simultaneously, H gasses could be incorporated as Fe-H alloys, increasing the H-rich inner core. Furthermore, because Mercury, Mars, and Earth’s moon do not have inner cores with high pressures ~364 GPa and Venus does not have enough H^13^ or D, these terrestrial planets do not support nuclear fusion, resulting in no evidence of plate tectonics.

## Conclusions

We provide a possible model for the origin of thermal energy from Earth’s interior without harmful radioactive wastes in which heat generation is the result of three-body nuclear fusion of deuterons confined within hexagonal FeDx core-centre crystals: ^2^D + ^2^D + ^2^D → 2^1^H + ^4^He + 2 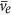 + 20.85 MeV. The KamLAND Collaboration was able to observe anti-neutrinos by nuclear fusion in the D-rich inner core, provided that scientists at KamLAND could believe the nuclear fusion.

## Additional Information

**How to cite this article**: Fukuhara, M. Possible generation of heat from nuclear fusion in Earth’s inner core. *Sci. Rep.* **6**, 37740; doi: 10.1038/srep37740 (2016).

**Publisher's note:** Springer Nature remains neutral with regard to jurisdictional claims in published maps and institutional affiliations.

## Supplementary Material

Supplementary Information
