# Corrigendum: Possible generation of heat from nuclear fusion in Earth’s inner core

**DOI:** 10.1038/srep46436

**Published:** 2017-05-02

**Authors:** Mikio Fukuhara

Scientific Reports 6: Article number: 3774010.1038/srep37740; published online 11 23 2016; updated on 05 02 2017

The original version of this Article contained errors resulting from the incorrect calculation of formation energy in Equation 6. In the Abstract,

“^2^D + ^2^D + ^2^D → 2^1^H + ^4^He + 2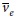 + 20. 85 MeV. The possible heat generation rate can generation rate can be calculated as 8.12 × 10^12^ J/m^3^”.

Now reads:

“^2^D + ^2^D + ^2^D → 2^1^H + ^4^He + e^-^ + 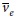 + 21. 63 MeV. The possible heat generation rate can generation rate can be calculated as 1.27 × 10^15^ J/m^3^”.

In addition, there was an error in the ‘Introduction section’, where

“Increase in paraeomagnetic magnitude between 2.7–2.1 billion years”

Now reads:

“Increase in palaeomagnetic magnitude between 2.7–2.1 billion years”

There were also errors in Equations 6, 7, 9, 10 and 11, which now read:


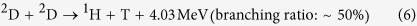



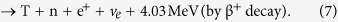



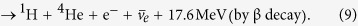



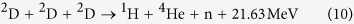



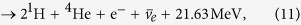


There were also errors in Equations 23, 24, 25 and 26 which now read:


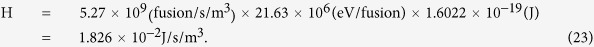



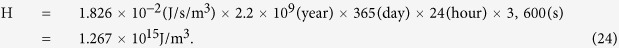



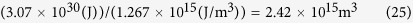



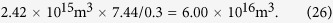


Under the sub-heading “Existence of a D-rich inner core”,

“On the other hand, because the incorporated gross weight of D from the creation of Earth’s core to present time is estimated as 6.67 × 10^21^ kg (=100 Mkm^3^ × 2 Mg/m^3^ × 4/20 × 1/0.006), the volume of Fe-D crystals up through the present is 4.99 × 10^17^ m^3^ (6.67 × 10^21^/13.36 [Mg/m^3^]), resulting in an unreacted (Fe-D) crystal volume of 4.37 × 10^17^ m^3^ (=4.99 × 10^17^ − 6.23 × 10^16^)”.

Now reads:

“On the other hand, because the incorporated gross weight of D from the creation of Earth’s core to present time is estimated as 6.67 × 10^21^ kg (=100 Mkm^3^ × 2 Mg/m^3^ × 4/20 × 1/0.006), the volume of Fe-D crystals up through the present is 4.99 × 10^17^ m^3^ (6.67 × 10^21^/13.36 [Mg/m^3^]), resulting in an unreacted (Fe-D) crystal volume of 4.39 × 10^17^ m^3^ (=4.99 × 10^17^ − 6.00 × 10^16^)”.

Finally, there was an error in the ‘Conclusions’ section where:


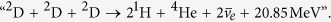


Now reads:


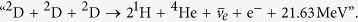


These errors have now been corrected in the HTML and PDF versions of this Article.

