# Supplementary material for: Possible generation of heat from nuclear fusion in Earth’s inner core
Source: Sci Rep. 2016 Nov 23;6:37740. doi: 10.1038/srep37740 (PMC5120317; doi:10.1038/srep37740)
Supplement: Supplementary Information [file srep37740-s1.pdf]

Supplementary Information

Mikio Fukuhara [1,2]

[1] New Industry Creation Hatchery Centre, Tohoku University, Sendai, Japan 980-8579
[2] Waseda University Research Organization for Nano & Life Innovation, Green Device Laboratory, Tokyo, Japan

Possible generation of heat from nuclear fusion in Earth's inner core

1. Geothermal gradient

High-pressure experimental studies, using laser-heated diamond-anvil cells based on X-ray diffraction and optical spectroscopies, have provided significant information for understanding Earth's structure. The temperature in Earth's interior has been reported to be a function of depth[19, 20, 48]. The depth dependency (**a**) of temperature is presented in Fig. 1S, along with estimated curves **b**, **c** and **d**, which are the temperature curves caused by radioactive decay at inside of the crust (6−40 km) and the mantle (410−2,900 km), as reported by the KamLAND Collaboration[10], and Earth's primordial heat supply, respectively. The curve **e** is a summation curve of curves **b**, **c** and **d**, which are interpolated using a linear allocation of heat contributions from the mantle (10 TW), the crust (7.9 TW) and Earth's primitive heat supply (26.3 TW) at Earth's surface, respectively. The slope characteristics of curve **e** are not coincident with the reported slope of **a**, which shows a negative linear slope. Thus, this fact severely constrains the possibility that radiogenic heat production in the crust and the mantle are producing $\bar{\nu}_e$.

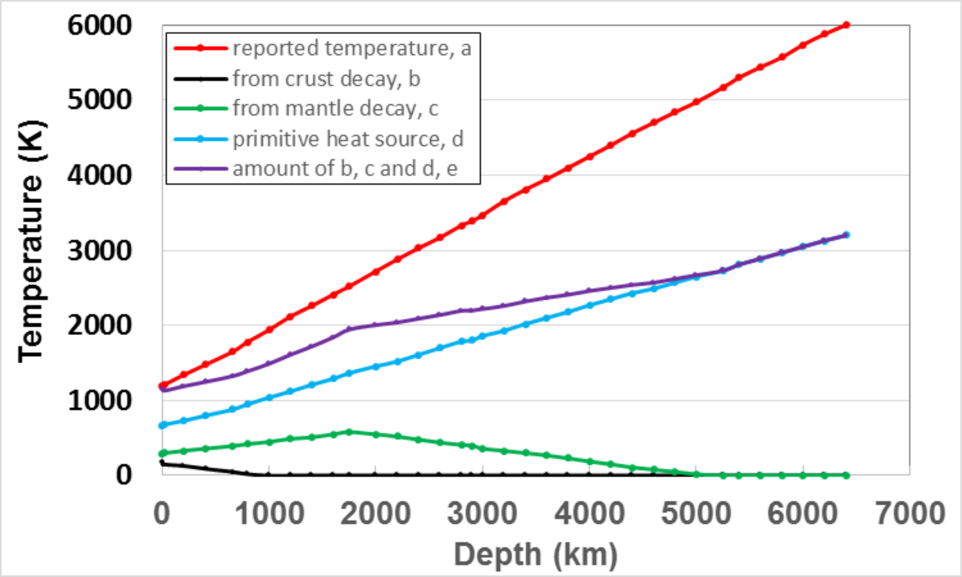

Fig. 1S The depth dependency of temperature in Earth's core. **a**: reported temperature curve. **b** and **c** : temperatures caused by radioactive decay at areas of the crust (6–40 km) and the mantle (410–2,900 km), respectively. **d**: temperature corresponding to Earth's primordial heat supply. **e**: summation curve of **b**, **c** and **d**.

2. Proton-mediated fusion reactions

Nuclear fusion is a reaction in which light nuclei are forced together under high temperature and ultra-high pressure conditions to produce heavier elements. For proton-mediated nuclear fusion, which is a cleaner and less hazardous reactions that does not produce high-energy neutrons, the following reactions are possible:

$$^{1}p + {}^{7}Li \rightarrow {}^{4}He + \alpha \quad (S1)$$

$$^{1}p + {}^{9}Be \rightarrow {}^{6}Li + \alpha \quad (S2)$$

$$^{1}p + {}^{11}B \rightarrow {}^{8}Be + \alpha \quad (S3)$$

$$^{1}p + {}^{11}C \rightarrow {}^{8}Be + \alpha \quad (S4)$$

$$^{1}p + {}^{13}C \rightarrow {}^{10}B + \alpha \quad (S5)$$

$$^{1}p + ^{13}N \rightarrow ^{10}B + \alpha \quad (S6)$$

$$^{1}p + ^{15}N \rightarrow ^{12}C + \alpha \quad (S7)$$

$$^{1}p + ^{17}O \rightarrow ^{14}N + \alpha \quad (S8)$$

$$^{1}p + ^{19}F \rightarrow ^{16}O + \alpha. \quad (S9)$$

Proton-mediated nuclear fusion is characterised by unstable reactional nuclei with odd atomic numbers. Because the existence of the reactional nuclei ^{11}C, ^{13}C, ^{13}N, ^{15}N and ^{17}O in the earth are 0, 1.1, 0, 0.37 and 0.038%, respectively, the possibility of Eqs. (S4), (S5), (S6), (S7) and (S8) are not possible and must be eliminated. On the other hand, although the amounts of stable reactional nuclei with odd numbers ^{7}Li, ^{9}Be, ^{11}B and ^{19}F are 92.5, 100, 80.1 and 100%, respectively, the possibility of Eqs. (S2) and (S3) must also be removed because the amounts of product nuclei ^{6}Li and ^{8}Be are 7.5 and 0%, respectively, in nature. Thus, only Eqs. (S1) and (S9) remain as candidates for proton-mediated fusion reactions.

3. Deuteron mediated fusion reactions

For deuteron-mediated nuclear fusion, which is a cleaner and less hazardous reaction that does not produce high-energy neutrons, the following reactions are possible:

$$^{2}D + ^{2}D \rightarrow ^{4}He \quad (S10)$$

$$^{2}D + ^{6}Li \rightarrow ^{4}He + \alpha \quad (S11)$$

$$^{2}D + ^{9}Be \rightarrow ^{7}Li + \alpha \quad (S12)$$

$$^{2}D + ^{11}B \rightarrow ^{9}Be + \alpha \quad (S13)$$

$$^{2}D + ^{12}C \rightarrow ^{10}B + \alpha \quad (S14)$$

$$^{2}D + ^{13}C \rightarrow ^{11}B + \alpha \quad (S15)$$

$$^{2}D + ^{14}N \rightarrow ^{12}C + \alpha \quad (S16)$$

$$^{2}D + ^{15}N \rightarrow ^{13}C + \alpha \quad (S17)$$

$$^{2}D + {}^{16}O \rightarrow {}^{14}N + \alpha \quad \text{(S18)}$$

$$^{2}D + {}^{17}O \rightarrow {}^{15}N + \alpha \quad \text{(S19)}$$

$$^{2}D + {}^{18}O \rightarrow {}^{16}O + \alpha \quad \text{(S20)}$$

However, because the amounts of reactional nuclei ^{6}Li, ^{13}C, ^{15}N, ^{17}O and ^{18}O in Earth are 7.5, 1.1, 0.366, 0.038 and 0.200%, respectively, the possibility of Eqs. (S11), (S15), (S17), (S19) and (S20) are not possible and must be eliminated. On the other hand, because the Clarke numbers of Li, Be and B are small, Eqs. (S12), (S13) and (S14) must be also removed. Thus, Eqs. (S10), (S16) and (S18) remain as the only candidates for deuteron-mediated fusion reactions.

4. Possible collective resonance and three-dimensional charge density wave

In the interaction mode of the Fe–D system, we note that the breathing-mode displacement (Fig. 2S) of the oxygen atoms in the perovskite solid solution $BaPb_{1-x}Bi_xO_3$ causes the alternating expansion and contraction of the oxygen octahedral around the nonequivalent Bi(I) and Bi(II) atoms[49] and leads to a complete charge disproportion (charge-density wave [CDW] instability) state, i.e., an alternating Bi^{3+}–Bi^{5+} array[33]. In this case, the Fermi surface nests perfectly and the situation is favourable for the formation of a CDW. The CDW doubles the unit cell and creates a gap near the Fermi level. Because the deuteron atoms exist as itinerant deuterons in the FeDx lattice, we expect a similar situation to occur for the FeDx.

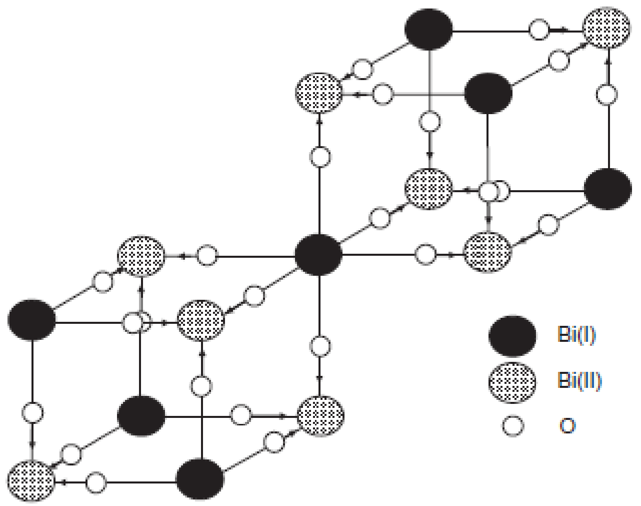

Fig. 2S Breathing mode distortions of O atoms that form alternating arrays of nonequivalent Bi(I)–Bi(II) sites.

5. Calculation of the reduced radius of D under high pressure

Based on an in situ X-ray study of the phase transition of Fe at multimegabar pressures[38], we obtained the density (13.3574 Mg/m^3) of hexagonal (ε) Fe at 364 GPa in the core centre. The radius of ε-Fe can calculated using Eq. (S21)

$$\left(\frac{\rho_1}{\rho_0}\right) = \left(\frac{R_0}{R_1}\right)^3, \qquad \text{(S21)}$$

where ρ_0 (7.87 M/m^3)and ρ_1 (13.3574 Mg/m^3) and R_0 (0.124 nm) and R_1 are the Fe densities and Fe radii at ambient pressure and 364 GPa, respectively. Because R_1 is obtained as 0.10395 nm from Eq. (S21), the radius r_1 of D squeezed in tetrahedral sites can be calculated as

$$2r_1 = 2 \times 0.10395 \times 0.225 \cong 0.0468 \text{ nm} \qquad \text{(S22)}$$

If the three-body reaction occurs at tetrahedral sites, we get 0.00484 nm as r_1, from the geometrical relation $\sqrt{2}\,(\sqrt{2}+2)\ r_1 = 0.225R_1$.

6. Physical role of neutral pions for the nuclear fusion of He

Regarding the role of neutral pions, the possible coexistence of an electron and an electron neutrino in the nucleus was reported, based on the weak interactions of β^--decay[50]. Provided that the electron of an atom takes its share of both electromagnetic and weak interactions according as the zone ratio, we can see that one electron and one neutrino exist in a proton and neutron, respectively. The electron and the neutrino are coupled as an *s*-wave boson in nucleus.

When a He atom is formed from two deuterons, quarks, electrons and neutrinos must be mediated by charged and neutral intermediated bosons, **W** and $\mathbf{Z}^0$ (Fig. 3S), respectively, resulting in mediation of charged and neutral pions.

$$u + e^- \leftrightarrow d + \nu \qquad \text{(S23)}$$

$$e^+ + \bar{\nu}_e \leftrightarrow e^- + \nu \qquad \text{(S24)}$$

$$e^- + \nu \leftrightarrow e^- + \nu \qquad \text{(S25)}$$

$$(Z, A) \leftrightarrow (Z+2, A) + 2\,e^- + 2\nu \qquad \text{(S26)}$$

From Fig. 2S, the double addition of Eq. (S25), i.e., two e^-–ν pairs is equivalent to an addition of neutral pions. The pions within the nucleus allow nucleonic species to bond and transmute with each other[36]. Because the neutral pion does not experience a Coulomb barrier, it can more easily enter within the effective nuclear force field of D-D pairs at close proximity compared to charged pions.

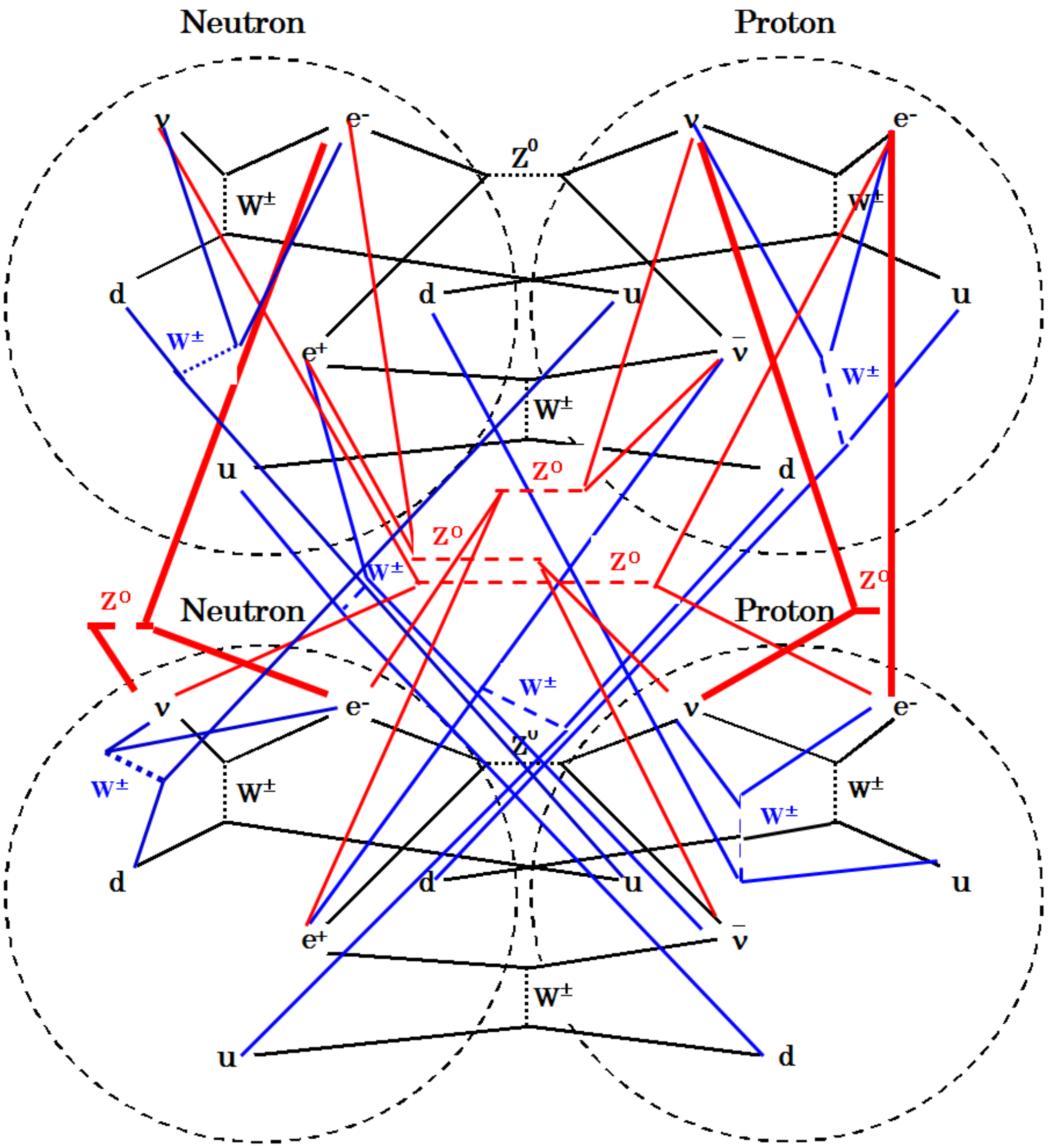

Fig. 3S Schematic representation of elementary particle interactions, **d**+ $\nu \leftrightarrow$ **u** + e^-, $e^+ + \bar{\nu}_e \leftrightarrow e^- + \nu$ and $e^- + \nu \leftrightarrow e^- + \nu$, mediated by **W** and $\mathbf{Z^0}$

bosons in protons and neutrons of a deuterium nucleus, respectively. **u** and **d** are up and down quarks, respectively. The black lines are **W** and $\mathbf{Z^0}$ mediated interaction between one proton and one neutron. **W** and $\mathbf{Z^0}$ mediated interactions among multiple protons and neutrons are shown with blue and red lines, respectively.

7. Emission of excited electrons

Seismic electromagnetic activities, radio wave emission, lightening, *etc.* are explained by the electrical response of rocks with and without quarts[51, 52]. In particular, exoelectron emission signals are associated with the stick-slip of solids along heat flows under high pressure[53, 54]. According to the free electron model by Zel'dovich[37] and Al'tshuler[55], on the other hand, metallic elements under pressures greater than 100 GPa near Earth's inner core centre disappear periodicity, which reflects the electronic shell structure of atoms. The outer and degenerated inner electrons act just like free electrons. This is called as "pressure ionisation".

8. Reaction rate of the activated state product

Because the transmutation is attained by instantaneous spontaneous fission of the activated state product $^2D^2D$ of the D-D reaction, we first consider the reaction rate for the product to be the transmutation rate.

In the Born-Oppenheimer approximation, the reaction rate R for the D-D reaction is proportional to the probability $|\Psi(0)|^2$, where Ψ is the wave function of the interacting D-D pair at the origin of the centre of mass coordinate system[56],

$$R = B\ |\Psi(0)|^2, \tag{S27}$$

where B is the nuclear reaction constant (cm^3/s). At very low energies, the cross section σ for the reaction can be written as[57]

$$\sigma = \frac{B}{V}\ H_0^2, \tag{S28}$$

where V is the relative velocity of the incident particle and H_0^2 is the s-wave Coulomb penetration factor. When the finite size of the interaction volume is

neglected, the Coulomb factor at low energies is

$$H_0^2 \cong 2\pi\ \eta_0 \exp(-\ 2\pi\eta_0) \tag{S29}$$

$$\eta_0\ = \frac{e^2}{\hbar V}\ , \tag{S30}$$

where η_0 is the Sommerfeld parameter. From Eqs. (S28), (S29) and (S26), we obtain the familiar Gamow formula[34]

$$\sigma = \frac{S}{E}\ 2\pi\eta_0\ \exp(-\ 2\pi\eta_0) = \frac{S}{E}\ \ exp\ (-\ \frac{\beta}{\sqrt{E}}) \tag{S31}$$

$$E = \frac{E_{m_2}}{E_{m_1}+E_{m_2}}, \tag{S32}$$

where S is the astrophysical S-factor, β is the Coulomb barrier tunnelling constant and E is the reduced energy of masses m_1 and m_2 of the incident particle and a target nucleus, respectively. Next, we introduce the neutral pions. Pions are responsible for all low-energy nuclear interactions and must be involved in this nuclear interaction[37]. If the neutral pion is provided by the emission of two exited electrons, the velocity V of the neutral pion is given by

$$\frac{1}{2} m_\pi^0 v_{\pi^0}{}^2 = 2\ \times\ \frac{1}{2} m_e (3 \times 10^6\ (Ref.57))^2, \tag{S33}$$

$$m_{\pi^0} = 268\, m_e\ = 135.0\, MeV, \tag{S34}$$

where m_{π^0} and m_e are the masses of the neutral pion and the electron, respectively. From Eqs. (S33) and (S34), we obtain v_{π^0} = 2.592 × 10^5 m/s. Here we consider the reaction D → n +H from Eq.(10). When the fast neutron n collides with H, we find that

$$\frac{1}{2} m_n v_n\ (1.36 \times 10^4 (Ref.45))^2 = \frac{1}{2} \mathrm{M}_D v_D{}^2 \tag{S35}$$

$$M_D = m_p + m_n, \tag{S36}$$

where M_D is the mass of deuteron. From Eq. (S35), we obtain v_D= 9,761.4 m/s. Next we consider the reaction D + D →He. When one neutral pion collides with one deuteron, as in Eq. (18), we find that

$$2 \times \frac{1}{2} m_{\pi^0} {v_{\pi^0}}^2 = \frac{1}{2} M' V_{M'}, \quad \text{(S37)}$$

$$M' = m_p + m_n + m_{\pi^\pm} + m_{\pi^0}, \quad \text{(S38)}$$

where $m_p, m_n\ and\ m_{\pi^\pm}$ are the masses of the proton (=938.27 MeV), neutron (=939.55 MeV) and charged pion (139.6 MeV), respectively, of the activated state product M', and $V_{M'}$ is velocity of M', provided that the mass deviation energy is negligible. $V_{M'}$ is calculated as

$$V_{M'} = 9.189 \times 10^4 \text{ m/s}. \quad \text{(S39)}$$

The third D further collides with M' in Eq. (11), yielding He and 2H.

$$\frac{1}{2} \times M_D {V_D}^2 = \frac{1}{2} \times M'' {V_{M''}}^2 \quad \text{(S40)}$$

$$M'' = m_p + m_n + m_{\pi^\pm} + m_{\pi^0} + 2m_p, \quad \text{(S41)}$$

where M" is the activated state product DDD of Eq. (11). Hence, the velocity of the reduced mass of DDD is

$$V_{M''} = 6{,}641 \text{ m/s}. \quad \text{(S42)}$$

To obtain the Coulomb factor H_0^2 in Eq. (S30), we must calculate η_0. Using Eq. (S42), we get

$$\eta_0 = \frac{e^2}{\hbar V_{M'}} = 329.7 \quad \text{(S43)}$$

Then we consider the screening effect of the Coulomb repulsion to enhance the fusion probability for deuteron pairs. At very high pressures in excess of 0.5 Mg/cm^3, the individual electron shell structures of the atoms vanish and are replaced by a statistical distribution of the electrons in the field of the atomic nuclei [37]. Such electrons in the outer shell behave as if they were free particles. As is assumed for compacted matter under high pressure, the total

number of oscillating electrons in collective resonance for Fe is ~8. Since the screening by many electrons can be apparently treated as one electron with the total mass of all electrons involved, the effective change of deuteron pairs is reduced to e/8 by the electron charge screening effect. This means

$$\eta = \frac{1}{8}\eta_0 = 41.21 \tag{S44}$$

From Eqs. (S29) and (S44), we get

$$H_0^2 = 2\pi \times 41.21 \exp(-2\pi \times 41.21)$$

$$= 1.756 \times 10^{-13}. \tag{S45}$$

Next we consider B in Eq. (S27). Here it should be noted that Jackson[58] has reported that the negative meson should be able to act as a catalyst many times during its lifetime, and the reaction rate for the D-D reaction in the mesonic molecule is on the order of 10^6 times that of the observed rate[59]. For this reason, the meson is quite effective at lowering the Coulomb barrier between two nuclei by virtue of its very small orbit around one of them, because the mesonic atom acts as a neutron in its penetration of another nucleus. Hence, by analogy we infer that the rate can be treated as $B = 2 \times 10^{-8}$ cm^3/s, based on the catalytic help of neutral pions. Thus we get

$$\sigma = \frac{2.0 \times 10^{-8} \times 1.756 \times 10^{-13}}{6.641 \times 10^5}$$

$$= 5.288 \times 10^{-28}\ \mathrm{cm}^2 = 5.288 \times 10^{-4}\ \mathrm{barn} \tag{S46}$$

When we consider a pion exchange force between nucleons, we assume that the S factor is enhanced as a function of the force[42]. We apply the effect of the charged pions to that of the neutral pion. Because the addition of two neutral pions increases the S factor (=10^{11} keV barn) by four orders of magnitude with respect to the conventional condition of two charged pions, Eq. (S31) gives

$$E = \frac{10^{11}\ (keV\ barn)}{5.288 \times 10^4\ (barn)} \times 1.756 \times 10^{-13}$$

$$= 33.245 \text{ eV} \quad \text{(S47)}$$

This value is plausible for neutral pion catalyzed nuclear fusion within a D-D-D system. Substituting σ = 5.288 × 10^{-4} barn, S = 10^{11} keV barn for the D-D-D reaction and E = 0.0332 keV into Eq. (S31) gives the Coulomb barrier tunnelling constant

$$\beta = 209.2 \text{ eV}. \quad \text{(S48)}$$

Thus, we have

$$\sigma = \frac{10^{11}}{E}\exp\left(\frac{-209.2}{\sqrt{E}}\right) \ . \quad \text{(S49)}$$

The nuclear reaction rate/cm^3 is given by

$$R = N_D N_{coh} V_{M''}\,\sigma \quad \text{(S50)}$$

where N_D is the deuteron number of density, and N_{coh} is the multiplicity factor according to lattice-site conditions. Because N_D increases as the inverse of the cubic function of the radius ratio $(2r_3/2r_1)^3 = (0.02075/0.074)^3$ = 1/45.4, we use $N_D = 5 \times 10^{23}$/cm^3. If N_{coh} = 30/cm^3 (Ref. 59), we obtain

$$R = 5 \times 10^{23}\ (1/\text{cm}^3) \times 30(1/\text{cm}^3) \times 6.64 \times 10^5\ (\text{cm/s}) \times 5.288 \text{ x } 10^{-28}\ (\text{cm}^2)$$

$$= 5266.8 \text{ fusion/s/cm}^3$$

$$\cong 5.3 \times 10^9 \text{ fusion/s/m}^3 \quad \text{(S51)}$$

References

48. Ritter, J. R. R. Rising through Earth's mantle, *Science*, **286**, 1865-1866 (1999)

49. Mattheiss, H. & Hamman, R. D. Electronic Structure of $BaPb_{1-x}Bi_xO_3$, *Phys. Rev.* B**28**, 4227-4241 (1983).

50. Fukuhara, M. Possible coupled electron and electron neutrino in nucleus and its physical catalysis effect on D-D cold fusion into helium in Pd, Condensed Matter Nuclear Science, ed. by Takahasi et al., 547-554 (World Sci. Pub., London, 2006).

51. Enomoto Y. & Hashimoto, H. Emission of charged particles from indentation fracture of rocks, *Nature*, **346**, 641-643 (1990).

52. Freund, F. T., Takeuchi, A., Lau, B. W. S., Al-Manaseer, A., Fu, C. C., Bryant, N. A. & Ouzounov, D. Stimulated infrared emission from rocks: assessing a stress indicator, *eEarth Discuss.*,**1**, 97-121 (2006), www.electronic-earth-discuss.net /1/97/2006/.

53. Brune, J. N., Brown, S. & Johnson, P. A. Rupture mechanism and interface separation in foam rubber models of earthquakes: a possible solution to the heat flow paradox and the paradox of large overthrusts, *Technophysics*, **218**, 59-67 (1993).

54. Tsutsumi, A. & Shirai, N. Electromagnetic signals associated with stick-slip of quartz-free rocks, *Tectonophysics*, **450**, 79-84 (2008).

55. Al'tshuler, L. V. & Bakanova, A. A. Electronic structure and compressibility of metals at high pressures, *Soviet. Phys.* –Usp, **11**, 678-689 (1969).

56. Van Siclen, C.D. & Jones, S. E. Piezonuclear fusion in isotopic hydrogen molecules. *J. Phys.* G, **12**, 213-221 (1986).

57. Serway, R. A. Physics for Science and Engineers with Modern Physics, 3rd. ed., p.645, (Saunders College Publishing, Philadelphia, 1990).
58. Jackson, J. D. Catalysis of nuclear reactions between hydrogen isotopes by μ^- mesons, *Phys. Rev.* **106**, 330-339 (1957).
59. Takahashi, A., Iida, T., Miyamaru, H., Multibody fusion model to explain experimental results. *Fus. Tech.* **27**, 71-85 (1995).
